# Versatile Toolbox for High Throughput Biochemical and Functional Studies with Fluorescent Fusion Proteins

**DOI:** 10.1371/journal.pone.0036967

**Published:** 2012-05-11

**Authors:** Garwin Pichler, Antonia Jack, Patricia Wolf, Sandra B. Hake

**Affiliations:** 1 Department of Biology II and Center for Integrated Protein Science Munich (CIPSM), Ludwig Maximilians University Munich, Planegg-Martinsried, Munich, Germany; 2 Center for Integrated Protein Science Munich at the Adolf-Butenandt Institute, Department of Molecular Biology, Ludwig Maximilians University of Munich, Munich, Germany; Université Paris-Diderot, France

## Abstract

Fluorescent fusion proteins are widely used to study protein localization and interaction dynamics in living cells. However, to fully characterize proteins and to understand their function it is crucial to determine biochemical characteristics such as enzymatic activity and binding specificity. Here we demonstrate an easy, reliable and versatile medium/high-throughput method to study biochemical and functional characteristics of fluorescent fusion proteins. Using a new system based on 96-well micro plates comprising an immobilized GFP-binding protein (GFP-mulitTrap), we performed fast and efficient one-step purification of different GFP- and YFP-fusion proteins from crude cell lysate. After immobilization we determined highly reproducible binding ratios of cellular expressed GFP-fusion proteins to histone-tail peptides, DNA or selected RFP-fusion proteins. In particular, we found Cbx1 preferentially binding to di-and trimethylated H3K9 that is abolished by phosphorylation of the adjacent serine. DNA binding assays showed, that the MBD domain of MeCP2 discriminates between fully methylated over unmethylated DNA and protein-protein interactions studies demonstrate, that the PBD domain of Dnmt1 is essential for binding to PCNA. Moreover, using an ELISA-based approach, we detected endogenous PCNA and histone H3 bound at GFP-fusions. In addition, we quantified the level of H3K4me2 on nucleosomes containing different histone variants. In summary, we present an innovative medium/high-throughput approach to analyse binding specificities of fluroescently labeled fusion proteins and to detect endogenous interacting factors in a fast and reliable manner *in vitro*.

## Introduction

Over the past decade a variety of proteomic approaches have been used to identify cellular components in order to understand the mechanism and inner workings of cells [1]. For example, mass spectrometry-based proteomics uncovered the proteome of many different organisms as well as cell-type specific differences in protein expression. However, to understand and characterize the function of single proteins, as well as the interplay between different factors, it is essential to gain further insights into their abundance, localization, dynamic interactions and substrate specificities.

Fluorescent proteins like the green fluorescent proteins (GFP) [2] and spectral variants have become popular tools to study the localization and dynamic interactions of proteins *in vivo.* Despite, the availability of a variety of commercial mono- and polyclonal antibodies against GFP and other fluorescent proteins [3], [4] (e.g. Abcam, UK; Sigma, USA; Roche, Germany, ChromoTek, Germany), proteins are mostly fused to a small epitope tag such as FLAG or c-Myc to analyze biochemical characteristics like enzymatic activities and/or binding specificities. Thus, integration of such *in vitro* data with *in vivo* data obtained with fluorescently labeled proteins has, in part, been impeded by the simple fact that different protein tags are used for different applications. The gold standard to examine binding affinities is surface plasmon resonance (SPR) [5]. One drawback of this method is the need of large amount of proteins. Such proteins have to be expressed and purified from bacterial systems (e.g. *E.coli*) or lower eukaryotes such as yeast (e.g. *S. cerevisiae*). Thus, the recombinant proteins lack essential post-translational modifications or are not folded properly possibly leading to different binding properties and inaccurate results. In addition with SPR measurements one can only determine the binding affinity to one substrate. This does not reflect the *in vivo* situation where most proteins have the choice between many different binding substrates in parallel.

Protein microarrays are an alternative to study protein-protein interactions in high-throughput manner [6]. Once more the drawback of this *in vitro* method is the laborative and time-consuming preparation of recombinant proteins or protein domains. Therefore protein microarrays are limited to domains that can be produced as soluble, well-folded proteins [6].

Recently, specific GFP binding proteins based on single domain antibodies derived from Lama alpaca have been described [7] (GFP-Trap ChromoTek, Germany). The GFP-Trap exclusively binds to wtGFP, eGFP and GFP^S65T^ as well as to YFP and eYFP. Coupling to matrices including agarose beads or magnetic particles the GFP-Trap allows for one-step purification of GFP-fusion proteins. Previous studies made use of the GFP-Trap to perform a broad range of different methods including mass spectrometry analysis [8], DNA binding, DNA methyltransferase activity assays [9], as-well-as histone-tail peptide binding assays [10]. One mayor disadvantage of the GFP-Trap is, that batch purification of GFP-fusions is very laborious and time-consuming and one cannot test different GFP-fusion and/or assay conditions in parallel. Here, we present an innovative and versatile high-throughput method to quantitatively measure binding specificities and to detect endogenous interacting factors in a fast and reliable manner *in vitro*: 96-well micro plates coated with immobilized GFP-Trap (GFP-multiTrap). To demonstrate the general suitability of our assays, we choose already known binding partners and compared our results with previous publications. Using this method, we could confirm that Cbx1 preferentially binds to di- and trimethylated histone H3 lysine 9 and that this binding is abolished by phosphorylation of the adjacent serine 10 [11]–[13]. In addition, we determined a 4-fold preference of the MBD domain of MeCP2 for fully over unmethylated DNA in accordance to [14]–[16]. Furthermore, we performed protein-protein interaction assays and found that the Dnmt1 binds to PCNA in a PBD domain-dependent manner consistent to [17], [18]. In contrast, LigaseIII binds Xrcc1 but does not interact with PCNA [19], [20]. Using an ELISA-based assay, we were able to detect endogenous PCNA bound to immunoprecipiated Dnmt1, Fen1 and PCNA itself. In accordance with our protein-protein interaction data, Dnmt1 lacking the PBD domain (Dnmt1ΔPBD) could not co-immunoprecipate with PCNA. Consistent with our histone-tail peptide binding data, we could detect endogenous histone H3 bound to Cbx1. Finally, we quantified specific histone modifcations on nucleosomes comprising different histone variants. All of these data clearly demonstrate the versatility and easy handling of this high-troughput approach and its immense benefit to many researchers.

## Results

### One-step Purification of GFP-fusion Proteins

**Figure 1:**
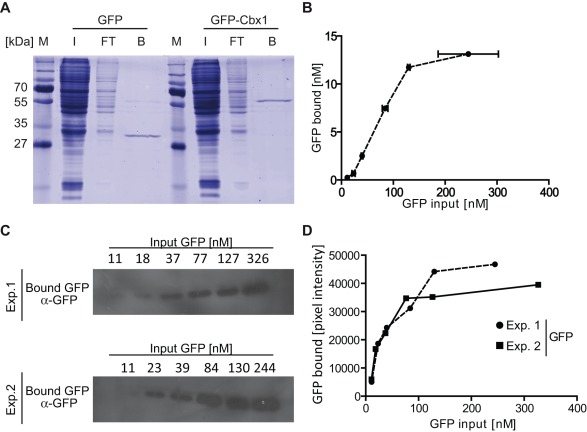
One-step purification of GFP and GFP-fusion proteins. Purification of GFP and GFP-Cbx1 expressed in HEK293T cells. All GFP concentrations were quantified via plate reader. (**A**) Purification of GFP and GFP-Cbx1 from HEK293T cell extracts, transciently transfected with the GFP-fusions. Input (I), flow-through (FT) and bound (B) fractions were separated by SDS-PAGE and visualized by coomassie staining. (**B**) Different amounts of GFP cell lysate were added into wells of a 96-well plate immobilized with the GFP-Trap (GFP-multiTrap).Shown are means ± SD from two independent experiments. (**C**) Bound GFP fractions from both independent experiments (B) were eluted, seperated by SDS-PAGE and visualized by immunoblot analysis using an anti-GFP mouse antibody (Roche, Germany). (**D**) Quantification of bound GFP fractions by immunoblotting. The mean intensities of the GFP signals were measured using Image J.

In a first step, we tested the efficiency of the GFP-multiTrap to purify GFP-fusion proteins from cellular extracts. First, we examined the pull-down efficiency of a GFP-tagged protein and chose GFP-Cbx1 as a model protein. Cbx1 is a chromodomain-containing protein related to the Drosophila HP1β, a well-studied heterochromatin-associated protein [11]. We used cell extracts from HEK293T cells transiently expressing GFP-Cbx1 or GFP, purified the GFP-fusions using the GFP-multiTrap, eluted the bound fractions, separated them by SDS-PAGE and visualized the bound proteins by coomassie staining. The bound fractions displayed mainly GFP as well as GFP-Cbx1 with only minor impurities (Figure 1A), providing therefore a reliable tool for downstream biochemical analyses. Notably, the washing conditions can be varied according to the downstream applications. In addition to these qualitative results, we performed experiments to quantify the pull-down efficiency. For this purpose we quantified the amount of bound GFP with varying concentrations of input GFP from cellular extracts. After binding, the single wells were subjected to several washing steps and bound GFP was analyzed by fluorescent read-out using a micro plate reader. Notably, the input amount of protein/substrate was measured in solution, whereas the bound fraction represents one value on the 96-well surface. We measured the fluorescence intensities of bound GFP and plotted the amount of bound GFP as a function of total GFP (Figure 1B). The amount of bound GFP increased linearly from 10 to 130 nM of total input and saturated between 130 and 400 nM. Next, we quantified the amount of bound GFP by immunoblotting. Therefore, we eluted the bound GFP fractions, separated them by SDS-PAGE, visualized the bound proteins by immunoblot analysis (Figure 1C) and quantified the GFP signal by measuring the mean intensity via Image J (Figure 1D). Similar to the quantifcation by fluorescent read out using a micro plate reader, the amount of bound GFP increases linearly from 10 to 130 nM of total input and saturates between 130 and 400 nM.

In summary, we demonstrated that the GFP-multiTrap allows for fast and efficient one-step purification of GFP-fusion proteins directly from crude cell lysates in a high-throughput manner. The method works well for both qualitative and quantitative measurements and the immunoprecipitated GFP-fusions can then be further tested in biochemical assays.

### In vitro Histone-tail Peptide and DNA Binding Assay

**Figure 2:**
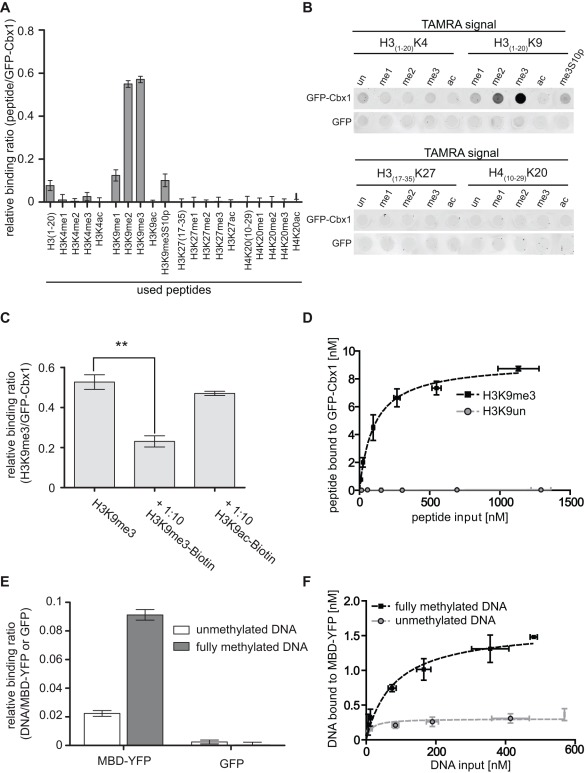
In vitro histone-tail peptide and DNA binding assay. *In vitro* binding ratios of fluorescently labeled substrates over bound GFP fusion proteins were determined. (A)–(D) *In vitro* histone-tail peptide binding assay with GFP-Cbx1. (**A**) Histone H3- and H4-tail binding specificities of Cbx1. A final concentration of 0.15 µM TAMRA-labeled histone-tail peptide was added per well. Fluorescent signals of bound TAMRA-labeled histone-tail peptides and GFP-fusion protein were quantified via plate reader. Shown are means ± SD from three independent experiments (**B**) Fluorescent signals of bound TAMRA-labeled histone-tail peptides visualized by fluorescent scanner. (**C**) Competition assay between TAMRA-labeled H3K9me3 and biotinylated histone-tail peptides with GFP-Cbx1. Shown are means ± SD from three independent experiments. Statistical significance between the binding ratios is indicated; **P<0.003. (**D**) Different amounts of TAMRA-labeled H3K9me3 and H3 histone-tail peptides were added to GFP-Cbx1. Three or two independent experiments for H3K9me3 or H3 histone-tail peptides were performed, respectively. Shown are means ± SD and the amount of bound histone-tail peptide was plotted as a function of total histone-tail peptide. The curve was fitted using GraphPad Prism and nonlinear regression. All input and bound fractions were quantified via a plate reader. (**E**) DNA binding specificities of the MBD domain of MeCP2 to un- and fully methylated DNA in direct competition. Shown are means ± SD from three independent experiments. (**F**) Different amounts of Atto550-labeled unmethylated and Atto700-labeled fully methylated DNA in direct competition were added to purified MBD-YFP. Shown are means ± SD from three independent experiments. The amount of bound DNA peptide was plotted as a function of total DNA. The curve was fitted using GraphPad Prism and nonlinear regression. All input and bound fractions were quantified via a plate reader.

In the next assay we determined whether this approach is also feasible to quantify binding affinities between GFP-proteins and peptides or DNA. First, we analyzed histone-tail peptide binding specificities of the chromobox homolog 1, Cbx1, fused with a N-terminal GFP-tag using the GFP-multiTrap. GFP-Cbx1 was purified from mammalian cell lysate, as described above, and the bound protein was incubated with TAMRA-labeled histone-tail peptides. A set of 20 different histone-tail peptides (Table 1) was used in technical triplicates in parallel and GFP served as negative control (GFP data is not shown). After removal of unbound substrate the amounts of protein and histone-tail peptide were determined by fluorescence intensity measurements using a micro plate reader. Binding ratios were calculated by dividing the concentration of bound histone-tail peptide by the concentration of GFP fusion (Figure 2A). GFP-Cbx1 preferentially binds H3K9me3 and H3K9me2 histone-tail peptides consistent with previous studies [11], [12]. As expected, the phosphorylation of serine 10 (S10p) next to the trimethylated lysine 9 leads prevents binding of Cbx1, which is in accordance with previous reports [13]. In addition to fluorescent quantification via a micro plate reader, we scanned the TAMRA signals using a Typhoon scanner (Figure 2B). Here, we detected TAMRA signals in the wells corresponding to di- and trimethylated H3K9. Notably, we did not detect differences in binding towards di-and trimethylated H3K9 using a micro plate reader. However, we could detect a preference for tri- over dimethylated H3K9 using a fluorescence scanner. These differences could result from different sensitivities of both methods. Furthermore, we performed a competition assay to demonstrate the specificity of the histone-tail peptide-binding assay. We incubated GFP-Cbx1 with TAMRA-labeled H3K9me3 in parallel with either biotinylated H3K9me3 or H3K9ac histone-tail peptides. As expected, the addition of biotinylated H3K9me3 histone-tail peptide significantly decreased the binding of Cbx1 to TAMRA-labeled H3K9me3, whereas the addition of biotinylated H3K9ac did not alter the binding ratios (Figure 2C). In previous studies [11], [12], the binding affinities of the HP1β chromo domain, the Drosophila homolog of mammalian Cbx1, for both di- and trimethylated H3K9 peptides have been found to be 7 and 2.5 µM, respectively. In contrast, we could not detect a significant difference in binding ratios between di- and trimethylated H3K9 histone tail peptides using a micro plate reader (Figure 2A). One explanation could be the use of different expression systems. While the binding ratios for the HP1β chromo domain were determined using bacterially expressed protein we used a fluorescent fusion protein derived from mammalian cells. In this context a recent study revealed that recombinant HP1α prepared from mammalian cultured cells exhibited a stronger binding affinity for K9-methylated histone H3 (H3K9me) in comparison to protein produced in *Escherichia coli* [21]. Biochemical analyses revealed that HP1α was multiply phosphorylated at N-terminal serine residues (S11–14) in human and mouse cells and that this phosphorylation enhanced the affinity of HP1α for H3K9me, displaying the importance of post-translational modifications for binding affinities [21]. To determine the binding affinity of GFP-Cbx1 to H3K9me3, we varied the input amount of histone-tail peptide. We plotted the amount of bound histone-tail peptide as a function of total peptide and fitted the values using GraphPad Prism and nonlinear regression (Figure 2D). The amount of bound H3K9me3 histone-tail peptide increases linearly and saturates at approximately 500 nM of input peptide. In contrast to H3K9me3, we could not detect any binding of Cbx1 to H3 histone-tail peptides. Notably, the exact determination of binding affinities was not possible due to differences in the technical measurement of input versus bound fractions. Here, the input amount of protein/substrate was measured in solution, whereas the bound fraction represents one value on the 96-well surface.

**Table 1:** Sequences of DNA oligonucleotides and histone-tail peptides.

DNA oligos							
DNA substrate	DNA sequence						DNA labeling
CG-up	5′- CTCAACAACTAACTACCATCCGGACCAGAAGAGTCATCATGG -3′						No
MG-up	5′- CTCAACAACTAACTACCATCMGGACCAGAAGAGTCATCATGG -3′						No
um550	5′- CCATGATGACTCTTCTGGTCCGGATGGTAGTTAGTTGTTGAG -3′						ATTO550 at 5′end
um700	5′- CCATGATGACTCTTCTGGTCCGGATGGTAGTTAGTTGTTGAG -3′						ATTO700 at 5′end
mC700	5′- CCATGATGACTCTTCTGGTCMGGATGGTAGTTAGTTGTTGAG -3′						ATTO700 at 5′end
**DNA substrates**							
**DNA substrate**	**CpG site**		**Label**		**Oligo I**		**Oligo II**
UMB-550	unmethylated		550		CG-up		um550
UMB-700	unmethylated		700		CG-up		um700
FMB-700	Fully methylated		700		MG-up		mC700
**DNA sets**							
**Binding set**				**Control set**			
UMB-550				UMB-550			
FMB-700				UMB-700			
**Histone-tail peptides**							
H3 (1–20)		ART K QTARKSTGGKAPRKQLK				TAMRA at C-terminus	
H3K4me1		ART X1 QTARKSTGGKAPRKQLK					
H3K4me2		ART X2 QTARKSTGGKAPRKQLK					
H3K4me3		ART X3 QTARKSTGGKAPRKQLK					
H3K4ac		ART Z QTARKSTGGKAPRKQLK					
H3K9me1		ARTKQTAR X1 S TGGKAPRKQLK					
H3K9me2		ARTKQTAR X2 S TGGKAPRKQLK					
H3K9me3		ARTKQTAR X3 S TGGKAPRKQLK					
H3K9me3S10p		ARTKQTAR X3 Z2 TGGKAPRKQLK					
H3K9ac		ARTKQTAR Z S TGGKAPRKQLK					
H3 (17–36)		RKQLATKAAR K SAPATGGVK				TAMRA at N-terminus	
H3K27me1		RKQLATKAAR X1 SAPATGGVK					
H3K27me2		RKQLATKAAR X2 SAPATGGVK					
H3K27me3		RKQLATKAAR X3 SAPATGGVK					
H3K27ac		RKQLATKAAR Z SAPATGGVK					
H4 (10–29)		LGKGGAKRHR K VLRDNIQGI					
H4K20me1		LGKGGAKRHR X1 VLRDNIQGI					
H4K20me2		LGKGGAKRHR X2 VLRDNIQGI					
H4K20me3		LGKGGAKRHR X3 VLRDNIQGI					
H4K20ac		LGKGGAKRHR Z VLRDNIQGI					

X1: monomethylated Lysine; X2: dimethylated Lysine; X3: trimethylated Lysine; Z: acetylated Lysine; Z2: phosphorylated Serine.

In addition to histone-tail peptide binding assays, we performed DNA-binding assays. We purified the methyl-binding domain (MBD) of MeCP2, fused with a C-terminal YFP tag, from cell extracts as described and performed competition binding analysis by incubating immobilized MBD-YFP with fluorescently labeled un- and fully methylated DNA (Table 1). As a result we observed a five-fold preference of MBD for fully methylated DNA over unmethylated DNA (Figure 2E). In addition, we measured the amount of bound DNA to MBD-YFP by varying the input amount of DNA. We plotted the amount of bound un- and fully methylated DNA as a function of total un-and fully methylated DNA and fitted the values using GraphPad Prism and nonlinear regression (Figure 2F). Similar to the relative binding ratios, MBD binds preferentially to fully methylated DNA. These results are in accordance with previous studies describing that MeCP2 interacts specifically with methylated DNA mediated by the MBD domain. In these studies, electrophoretic mobility shift assays (EMSA) using the isolated MBD domain expressed in *E. coli* were performed and dissociation constants of 14,7 and 1000 nM were calculated for symmetrically methylated and unmethylated DNA, respectively [14]–[16].

To assess the suitability of the *in vitro* histone-tail peptide and DNA binding assay for high-throughput applications, the Z-factor was calculated. For histone-tail peptide binding assays, we calculated the Z-factor using the relative binding ratios of H3K9me3 to GFP-Cbx1 as positive state and of H3K9me0 to GFP-Cbx1 as negative state. For the DNA binding assay, we calculated the Z-factor using the relative binding ratios of fully methylated DNA to MBD-YFP as positive state and of unmethylated DNA to MBD-YFP as negative state (Table 2). The Z-factors of 0.766 for the histone-tail peptide binding assay and 0.756 for the DNA binding assay strongly indicate that both assays are robust, reproducible and suitable for high-throughput applications.

**Table 2:** Overview of relative binding ratios and Z-factor values.

Relative binding ratios of Substrate/GFP- or YFP-fusion						
	Histone-tail peptide binding		DNA binding		Protein-protein binding	
Fusion protein	GFP-Cbx1		MBD-YFP		GFP-PBD	
Substrate	H3K9me3	H3K9un	Fully methylated DNA	Unmethylated DNA	RFP-PCNA	RFP
**Average ratio**	0,5715	0,0772	0,0912	0,0223	1,487	0,005
**Standard deviation**	0,0150	0,0236	0.0037	0.0019	0,2111	0,006
**Z-factor**	0,766		0.756		0.560	

Based on the average relative binding ratios and the standard deviations we calculated the Z-factor.

### In vitro Protein-protein Binding Assay

In addition to the detection of substrate specificity (e.g. histone-tail peptide) and DNA binding, analysis of the interaction with other cellular components and factors is essential to understand the function of proteins.

The use of fluorescence intensity read-out systems for the quantification of protein-protein interactions *in vitro* provides a new and simple method avoiding laborious and inaccurate protein detection using conventional immunoblotting systems.

**Figure 3:**
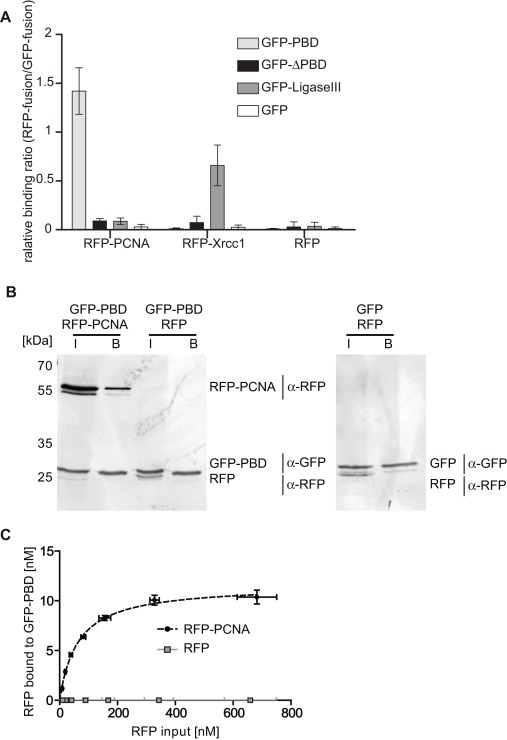
In vitro Protein-Protein binding assay. (**A**) *In vitro* binding ratios of RFP-fusion proteins over GFP-fusion proteins. Shown are means ± SD from six independent experiments. (**B**) After immunoprecipitation input (I) and bound (B) fractions were separated by SDS-PAGE and visualized by immunoblot analysis using the anti-GFP rat monoclonal antibody; 3H9, and the anti-RFP rat monoclonal antibody, 5F8 (both ChromoTek, Germany). GFP-PBD: 30 kDa; RFP-PCNA: 56 kDa; GFP: 28 kDa, RFP: 26 kDa. (**C**) Different amounts of RFP-fusion protein were added to purified GFP-PBD. Shown are means ± SD from two independent experiments. The amount of bound RFP was plotted as a function of total RFP. The curve was fitted using GraphPad Prism and nonlinear regression. All input and bound fractions were quantified via a plate reader.

To address the question if such interaction analysis can be performed in a multi-well format we analyzed the interaction of single GFP-fusions with RFP-fusion proteins expressed in mammalian cells. More precisely, we determined quantitative binding ratios between nuclear located proteins involved in DNA-replication (PCNA) [17], [18], DNA-methylation (Dnmt1) [22] as well as in DNA-repair (Xrcc1) [23]. As described, we immobilized GFP-fusions on the GFP-multiTrap and incubated them with cell lysate containing RFP-fusion proteins. After binding, we removed unbound material, measured the concentrations of RFP and GFP and calculated the molar binding ratios. Firstly, we determined the binding ratios of the green fluorescent PCNA-binding domain of Dnmt1 (GFP-PBD) to RFP-PCNA and used Dnmt1ΔPBD as a negative control. By measuring the fluorescent signal intensities we detected that RFP-PCNA binds to GFP-PBD in a molar ratio of 1.42±0.31 but not to Dnmt1ΔPBD (Figure 3A).

For a direct comparison we eluted the bound fractions, separated them by SDS-PAGE and visualized the proteins by immunoblotting (Figure 3B). Both, GFP-PBD and RFP-PCNA are detected in the input and bound fractions whereas RFP is not visible in the bound fraction of GFP-PBD (Figure 3B).

In addition, we measured the amount of bound RFP-fusion to GFP-PBD with varying the input amount of RFP-fusion. We plotted the amount of bound RFP-fusion as a function of total RFP-fusion and fitted the values using GraphPad Prism and nonlinear regression (Figure 3C). Similar to the relative binding ratios, GFP-PBD binds to RFP-PCNA but not to RFP.

These results are in accordance with previous findings that Dnmt1 associates with the replication machinery by directly binding to PCNA, a homotrimeric ring which serves as loading platform for replication factors, and that this binding depends on the PCNA-binding domain in the very N-terminus of Dnmt1 [17], [18]. In addition by determining the quantitative binding ratio between both partner proteins our approach provides a more detailed insight in the binding events occurring at the central loading platform of the DNA replication.

Secondly, we determined the molar binding ratio of GFP-Ligase III to RFP-Xrcc1. Xrcc1 binds in a molar ratio of 0.61±0.14 to Ligase III but did not bind to other proteins such as GFP-PBD, GFP-Dnmt1ΔPBD or GFP. Previous studies demonstrated that DNA Ligase III was recruited to DNA repair sites via its BRCT domain mediated interaction with Xrcc1 [19], [20].

For the protein-protein binding assays, we calculated the Z-factor using the molar binding ratios of RFP-PCNA to GFP-PBD as positive state and RFP to GFP-PBD as negative state (Table 2). The Z-factor of 0.56 indicated that the protein-protein binding assay is robust and reproducible.

In summary, we demonstrate a new quantitative and reliable high-throughput method to analyze protein-protein interactions using GFP- and RFP-fusion proteins.

### Enzyme-linked Immunosorbent Assay (ELISA)

**Figure 4:**
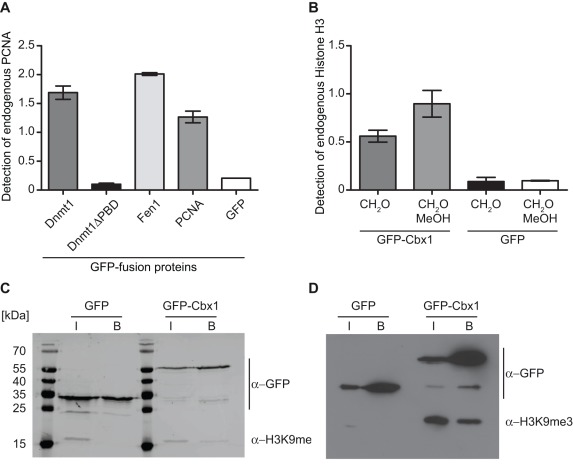
Pulldown of endogenous interaction partners. GFP-fusions were immunoprecipitated and endogenous interacting proteins were detected either by ELISA or immunoblot analysis. (**A**) ELISA signal (Absorbance at 450 nm) of bound endogenous PCNA detected by a PCNA antibody to purified GFP-fusion proteins. Shown are means ± SD from three independent experiments. (**B**) ELISA signal (Absorbance at 450 nm) of bound endogenous Histone H3 detected by an H3 antibody to purified GFP-fusion proteins. Bound fractions were either cross-linked with 2% formaldehyde (CH_2_O) and/or additionally permeabilized with MeOH. Shown are means ± SD from two independent experiments. (C) and (**D**) After immunoprecipitation input (I) and bound (B) fractions were separated by SDS-PAGE and visualized by immunoblot analysis. (**C**) The total protein concentration of the input fractions were adjusted. (**D**) The GFP concentrations of the input fractions were adjusted.

Next we examined endogenous protein-protein interactions using an ELISA assay. For this purpose, we precipitated GFP-fusion proteins in the 96 well format on the GFP-multiTrap and cross-linked bound fractions with formaldehyde (CH_2_O) and/or treated the bound fractions with methanol (MeOH). Using specific antibodies against PCNA, we determined the binding of endogenous PCNA to GFP fusions of Dnmt1, Dnmt1ΔPBD, PCNA, Fen1, which is a flap endonuclease and an essential DNA replication protein [24]. We could detect endogenous PCNA binding to Dnmt1 but not to Dnmt1ΔPBD similar to the results obtained with the protein-protein interaction assay using RFP-PCNA (Figure 4A). In addition, we detected binding of endogenous PCNA to Fen1 but also to PCNA itself. These results fit well to former studies showing that Fen1 or maturation factor 1 associates with PCNA in a stoichiometric complex of three Fen1 molecules per PCNA trimer [25], [26]. In addition to 100 described interacting partners, it is known that PCNA also interacts with itself and forms a trimeric ring, which is confirmed by our ELISA assay by giving a signal for endogenous PCNA binding to GFP-PCNA (Figure 4A).

Next, we determined the binding of Cbx1 to endogenous histone H3. Similar to PCNA, we precipitated GFP-Cbx1 and GFP and detected endogenous H3 via an H3-antibody coupled to HRP. In accordance with the experiments using TAMRA labeled histone 3 peptides, we observed an H3 ELISA signal for binding to Cbx1 but not to GFP. Using an H3K9me3-specific antibody, we could not detect an ELISA signal (data not shown), due to the fact that the tight binding of Cbx1 (Figure 2) to H3K9me3 most likely occludes the antibody epitope, as has been proposed for HP1 binding to H3K9me3. In this study, the histone H3 trimethyl-lysine epitope is embedded in an aromatic cage blocking thereby most likely the binding of any antibodies [27]. To further analyze the bound fractions, we eluted GFP-Cbx1 and GFP, separated them on an SDS-PAGE gel and visualized GFP and H3 by immunoblotting. Histone H3 was detectable in the input fractions of both GFP and GFP-Cbx1 but as expected, only in the bound fraction of GFP-Cbx1.

### Comparative Analysis of Posttranslational Histone Modifications

Histone posttranslational modifications play an important role in the structural organization of chromatin and often correlate to transcriptional activation or repression depending on their type and location. Recently, it has been shown that nucleosomal incorporation of histone variants can lead to alterations in modification patterning and that such changes may complement the properties brought by the variant itself [28].

**Figure 5:**
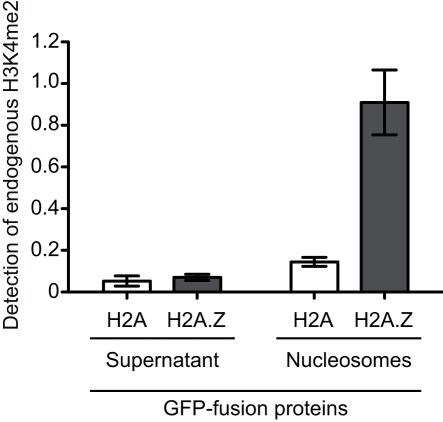
Comparative analysis of posttranslational histone modifications. Cytoplasmic supernatant (SN) or mononucleosome (MN) fractions prepared from HeLa cells expressing GFP-H2A or GFP-H2A.Z were precipitated and the levels of H3 and H3K4me2 were detected by ELISA (Absorbance at 450 nm). Shown are the H3K4me2 levels normalized to H3 and means ± SD from two independent experiments.

In order to investigate the suitability of the GFP-multiTrap in comparing such histone posttranslational modifications, we isolated nucleosomes from HeLa cells expressing either GFP-H2A or GFP-H2A.Z and precipitated them with the 96 well micro plate. GFP levels were then recorded (data not shown) to ensure equal loading of substrate per well. In addition, as a negative control, the cytoplasmic supernatant fraction was also incubated with the GFP-multiTrap. An ELISA approach was then used to quantify differences in histone H3K4me2 levels between the two different nucleosome compositions. Following cross-linking and permeablization, bound nucleosomes were incubated with either anti-H3, directly conjugated to HRP or anti-H3K4me2 (both antibodies Abcam, UK). Histone H3K4me2 levels were then normalized to the histone H3 signal. In accordance with published data, H2A containing nucleosomes were depleted in H3K4me2 where as those containing H2A.Z showed a large enrichment for this modification (Figure 5) [28].

## Discussion

One challenge of the proteomic era is the effective integration of proteomic, cell biological and biochemical data. Ideally, proteomic data on tissue and cell cycle-specific expression of specific proteins should be combined with subcellular localization and binding dynamics of fluorescent proteins. Additionally, it is crucial to determine cell biological and biochemical characteristics such as interacting factors, enzymatic activity and substrate binding specificities. The integration of all these different data has, in part, been impeded by the simple fact that different protein tags are used for different applications. Here, we present a new versatile, high-throughput method to determine *in vitro* binding specificities and to detect endogenous interacting factors of GFP-fusion proteins. We use 96-well micro plates with immobilized GFP-Trap (GFP-multiTrap) for fast and efficient purification of GFP-fusion proteins. We demonstrate the efficiency and purity of the GFP immunoprecipitation (Figure 1), a prerequisite to obtain reliable biochemical data on e.g. binding specificities. Moreover, we measured histone-tail binding, DNA and protein-protein binding ratios underlying the versatility of our approach (Figure 2 and 3 and Table 2). The suitability of the demonstrated assays for high-throughput biochemical and functional studies was assessed by calculating the Z-factors (Table 2). Therefore, our assay is suitable to examine an initial high-throughput screening for potential binding partners. Moreover, the assay can be used for compound screening. Additionally, our method allows for detection of endogenous interaction factors based on an ELISA assay (Figure 4 and 5).

In contrast to other high-throughput techniques like conventional microarrays, it does not require time-consuming recombinant protein expression and purification but allows for the direct biochemical analyses of GFP-fusion proteins expressed in mammalian cells. The versatile GFP-multiTrap combined with the widespread use of fluorescent fusion proteins now enables a fast and direct quantitative correlation of microscopic data concerning the subcellular localization and mobility of fluorescent fusion proteins with their enzymatic activity, interacting factors, and DNA binding properties combining cell biology and biochemistry with mutual benefits.

## Materials and Methods

### Expression Constructs, Cell Culture and Transfection

Mammalian expression constructs encoding GFP-Dnmt1, GFP-Dnmt1ΔPBD, GFP-PBD, GFP-PCNA, RFP-PCNA, GFP-Ligase III, mRFP, GFP, MBD-YFP, GFP-Fen1 and RFP-Xrcc1 were described previously [7], [20], [29]–[37]. Note that all constructs encode fusion proteins of GFP, RFP or yellow fluorescent protein (YFP). The Cbx1 expression construct was derived by PCR from mouse cDNA, cloned into pEGFP-C1 (Clontech, USA) and verified by DNA sequencing. Throughout this study enhanced GFP (eGFP) constructs were used and for simplicity referred to as GFP-fusions. HEK293T cells [30] and HeLa Kyoto [29] were cultured in DMEM supplemented with either 50 µg/ml gentamicin (HEK293T) or 1% penicillin/streptomycin (HeLa Kyoto) and 10% fetal calf serum. For expression of GFP/RFP/YFP fusion proteins, HEK293T cells were transfected with the corresponding expression constructs using polyethylenimine (Sigma, USA). 2. HeLa Kyoto cells were transfected using FuGene HD (Roche, Germany) according to the manufacturer’s instructions. The plasmid coding for GFP-H2A (H2A type 1, NP_003501.1) was kindly provided by Emily Bernstein (Mount Sinai Hospital) and the plasmid coding for GFP-Z-1 was a gift from Sachihiro Matsunaga (University of Tokyo). Stable cell lines were selected with 600 µg/ml G418 (PAA, Austria) and individual cell clones sorted by using a FACSAria machine (Becton Dickinson, Germany).

### Histone-tail Peptides and DNA Substrate Preparation

Fluorescently labeled DNA substrates were prepared by mixing two HPLC-purified DNA oligonucleotides (IBA GmbH, Germany Table 1) in equimolar amounts, denaturation for 30 sec at 92°C and slow cool-down to 25°C allowing hybridization. Histone-tail peptides were purchased as TAMRA conjugates and/or biotinylated (PSL, Germany) and are listed in Table 1.

### Preparation of Protein Extracts

HEK293T cells were cultured and transfected as described [38]. For extract preparation 1 mg/ml DNaseI, 1 mM PMSF and Protease Inhibitor cocktail (Roche, Germany) were included in the lysis buffer (20 mM Tris-HCl pH 7.5, 150 mM NaCl, 2 mM MgCl_2_, 0.5% NP40) or nuclear extract buffer (10 mM HEPES pH 7.9, 10 mM KCl, 1.5 mM MgCl_2_, 0.34 M Sucrose, 10%Glycerol, 1 mM β-mercapto-ethanol). Cells were lysed for 30 minutes on ice followed by a centrifugation step (15̀/12000 rpm/4°C). Extracts from transfected 10 cm plates were diluted to 500 µL with immunoprecipitation buffer (IP buffer; 20 mM Tris-HCl pH 7.5, 150 mM NaCl, 0.5 mM EDTA) or dilution buffer (20 mM HEPES pH 7.9, 150 mM KCl). An aliquot of 10 µL (2%) were added to SDS-containing sample buffer (referred to as Input (I)).

### Purification and Elution of GFP/YFP/RFP- Fusions

For purification, 100 µL or 50 µL precleared cellular lysate for full-area plates or half-area plates, respectively, was added per well and incubated for 2 hours at 4°C on a GFP-multiTrap plate by continuous shaking. After removing the supernatant, wells were washed twice with 100 µL of washing buffer (WB; 20 mM Tris-HCl pH 7.5, 100–300 mM NaCl, 0.5 mM EDTA) and 100 µL of IP or dilution buffer was added for measurement. The amounts of bound protein were determined by fluorescence intensity measurements with a Tecan Infinite M1000 plate reader (Tecan, Austria). Wavelengths for excitation and emission of GFP are 490±10 nm and 511±10 nm, for RFP are 586±5 nm and 608±10 nm and for YFP 525±5 nm and 538±5 nm, respectively. The concentration of proteins was calculated using calibration curves that were determined by measuring the fluorescence signal of known concentrations of purified GFP, RFP and YFP. Notably, factors interfering with fluorescence intensity measurements such as absorption of excitation light by cell lysates, auto fluorescence of the samples and/or scattering of the excitation/emission light by cell debris are negligible (Figure S1). Bound proteins were eluted with 300 mM Glycin pH 2.5 and subsequently buffered with 1 M Tris pH 7.5. Elution fractions were added to SDS-containing sample buffer (referred to as Bound (B)). Bound proteins were visualized by immunoblotting using the anti-GFP mouse monoclonal antibody (Roche, Germany).

### In vitro Histone-tail Peptide Binding Assay

The *in vitro* histone-tail binding assay was performed as described previously [10]. After one-step purification of GFP fusion proteins the wells were blocked with 100 µL 3% milk solved in TBS-T (0.075% Tween) for 30 minutes at 4°C on a plate vortex, shaking gently. After blocking, the wells were equilibrated in 50 µL IP buffer supplemented with 0.05% Tween. TAMRA-labeled histone-tail peptides were added either to a final concentration of 0.15 µM or of the indicated concentrations and the binding reaction was performed at RT for 20 min on a plate vortex, shaking gently. After removal of unbound substrate the amounts of protein and histone-tail peptide were determined by fluorescence intensity measurements. The concentrations of bound TAMRA-labeled histone-tail peptides were calculated using calibration curves that were determined by measuring a serial dilution of TAMRA-labeled peptides with known concentrations.

Binding ratios were calculated dividing the concentration of bound histone-tail peptide by the concentration of GFP fusion. Wavelengths for excitation and emission of TAMRA were 560±5 nm and 586±5 nm, respectively.

### In vitro DNA Binding Assay

*In vitro* DNA binding assay was performed as described previously [9], [10] with the following modifications. GFP/YFP fusions were purified from HEK293T extracts using the 96-well GFP-binder plates and incubated with two differentially labeled DNA substrates at a final concentration of either 100 nM or of the indicated concentration for 60 min at RT in IP buffer supplemented with 2 mM DTT and 100 ng/µL BSA. After removal of unbound substrate the amounts of protein and DNA were determined by fluorescence intensity measurements. The concentration of bound ATTO-labeled DNA substrates was calculated using calibration curves that were determined by measuring a serial dilution of DNA-coupled fluorophores with known concentrations. Binding ratios were calculated dividing the concentration of bound DNA substrate by the concentration of GFP/YFP fusion, corrected by values from a control experiment using DNA substrates of the same sequence but with different fluorescent label, and normalized by the total amount of bound DNA. Wavelengths for excitation and emission of ATTO550 were 545±5 nm and 575±5 nm and for ATTO700 700±10 nm and 720±10, respectively.

### Protein-Protein Interaction

GFP fusions were purified from HEK293T extracts using the 96-well GFP multiTrap plates, blocked with 3% milk and incubated with cellular extracts comprising the RFP fusions with the indicated concentrations for 30 min at RT. After removal of unbound RFP fusion (washing buffer) the amounts of proteins were determined by fluorescence intensity measurements. Binding ratios were calculated dividing the concentration of bound RFP fusion by the concentration of GFP fusion. Wavelengths for excitation and emission of RFP were 586±5 nm and 608±10 nm, respectively. Bound proteins were eluted and separated by SDS-PAGE and visualized by immunoblotting using the anti-GFP rat monoclonal antibody; 3H9, and the anti-red rat monoclonal antibody, 5F8 (both ChromoTek, Germany).

### Enzyme-linked Immunosorbent Assay (ELISA)

GFP fusions were purified (from HEK293T extracts) using the 96-well GFP-multiTrap plates and were washed twice with dilution buffer (for nucleosome experiments salt concentration was adjusted to 300 mM). After washing bound fractions were either cross-linked with 2% formaldehyde and/or additionally permeabilized with 100% MeOH. After blocking with 3% milk solved in TBS-T (0.075% Tween) the wells were incubated with primary antibody (monoclonal rat anti-H3-HRP (Abcam, UK), polyclonal rabbit anti-H3K4me2 (Abcam, UK) or monoclonal rat anti-PCNA, 16D10 (ChromoTek, Germany) overnight at 4°C on a plate vortex, shaking gently. The wells were washed three times with 200 µL TBS-T and horseradish peroxidase-conjugated secondary antibody (Sigma, USA) was incubated for 1 h at RT for the detection of PCNA or H3K4me2. The wells were washed again as described above. For PCNA experiments detection was carried out by incubating each well with 100 µL TMB (3,3′,5,5′-tetramethylbenzidine) for 10 minutes at RT. The reactions were stopped with the addition of 100 µL 1 M H_2_SO_4_. For nucleosome experiments, detection was carried out using OPD (Sigma, USA) according to the manufacturers instructions. Bound histone H3, PCNA or H3K4me2 levels were quantified by determination of the absorbance at 450 nm using a Tecan Infinite M1000 plate reader (Tecan, Austria).

### Preparation of Mononucleosomes

2×10^7^−10×10^7^ HeLa cells, expressing either GFP-H2A or GFP-H2A.Z, were incubated in PBS, 0.3% Triton X-100 and Protease Inhibitor Cocktail (Roche, Germany) for 10 min at 4°C. Nuclei were pelleted and supernatant (SN) transferred and retained. The pellet was washed once in PBS, resuspended in EX100 buffer (10 mM Hepes pH 7.6, 100 mM NaCl, 1.5 mM MgCl_2_, 0.5 mM EGTA, 10% (v/v) glycerol, 10 mM β-glycerol phosphate 1 mM DTT, Protease Inhibitor Cocktail (Roche, Germany)) and CaCl_2_ concentration adjusted to 2 mM. Resuspended nuclei were digested with 1.5 U MNase (Sigma, USA) for 20 min at 26°C. The reaction was stopped by addition of EGTA to a final concentration of 10 mM followed by centrifugation for 10 min at 1000 rcf, 4°C. Mononucleosome containing supernatant (MN) was retained.

### Calculation of the Z-factors

To assess the suitability of the assay for high-throughput biochemical and functional studies, the Z-factor was calculated using the equation 
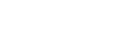
 [39]. In this equation, σ is the standard deviation of the positive (p) and the negative (n) control; μ is the mean value for the molar binding ratio (for positive (µ_p_) and negative (µ_n_) controls). The values of three independent experiments were used to calculate the Z-factor and all values are listed in Table 2.

## Supporting Information

Figure S1 **Factors interfering the measured fluorescence intensities.** (**A**) The concentrations of GFP and RFP expressed in HEK293T cells were measured in serial dilutions of crude cell extracts. Shown are means ± SD from two independent experiments. Fluorescence intensities were measured via a plate reader and the GFP and RFP concentrations were determined as described in the Material and Methods part. (**B**) Background GFP and RFP signals in cell lysates of untransfected HEK293T cells. The fluorescence intensities (FI) were measured via a plate reader and the concentrations were determined as described in the Material and Methods part.
(DOC)
