# Supplementary material for: Versatile Toolbox for High Throughput Biochemical and Functional Studies with Fluorescent Fusion Proteins
Source: PLoS One. 2012 May 11;7(5):e36967. doi: 10.1371/journal.pone.0036967 (PMC3350483; doi:10.1371/journal.pone.0036967)
Supplement: Figure S1 — Factors interfering the measured fluorescence intensities. (A) The concentrations of GFP and RFP expressed in HEK293T cells were measured in serial dilutions of crude cell extracts. Shown are means ± SD from two independent experiments. Fluorescence intensities were measured via a plate reader and the GFP and RFP concentrations were determined as described in the Material and Methods part. (B) Background GFP and RFP signals in cell lysates of untransfected HEK293T cells. The fluorescence intensities (FI) were measured via a plate reader and the concentrations were determined as described in the Material and Methods part. (DOC) [file pone.0036967.s001.doc]

**Supporting material**

**Figure S1: Factors interfering the measured fluorescence intensities.** (**A**) The concentrations of GFP and RFP expressed in HEK293T cells were measured in serial dilutions of crude cell extracts. Shown are means ± SD from two independent experiments. Fluorescence intensities were measured via a plate reader and the GFP and RFP concentrations were determined as described in the Material and Methods part. (**B**) Background GFP and RFP signals in cell lysates of untransfected HEK293T cells. The fluorescence intensities (FI) were measured via a plate reader and the concentrations were determined as described in the Material and Methods part.
